# Thoracic aorta vasoreactivity in rats under exhaustive exercise: effects of Lycium barbarum polysaccharides supplementation

**DOI:** 10.1186/1550-2783-10-47

**Published:** 2013-10-24

**Authors:** Zhifang Zhao, Yan Luo, Guanghua Li, Lingqin Zhu, Yin Wang, Xuehong Zhang

**Affiliations:** 1 Department of Physiology, School of Basic Medical Science, Ningxia Medical University, Yinchuan 750004, China; 2 Department of Pediatrics, General Hospital of Ningxia Medical University, Yinchuan 750004, China

**Keywords:** Lycium barbarum polysaccharides, Exhaustive exercise, Thoracic aorta, Vasoreactivity

## Abstract

**Background:**

Reduced arterial compliance is associated with an increased rate of morbidity and mortality in cardiovascular disease. Exercise is beneficial for compromised arterial compliance. However, the beneficial effects of exercise are lost with exhaustion. Lycium barbarum L. has been used in China for centuries to maintain good health. In this regard, the primary purpose of this study was to characterize the effects of the polysaccharides from Lycium barbarum (LBPs) on arterial compliance during exhaustive exercise.

**Methods:**

A four-week swimming exercise program was designed for rats, and the blood levels of malondialdehyde (MDA), super oxide dismutase (SOD), nitric oxide(NO) and heat shock protein 70(HSP70) were detected. The tension of aorta rings was measured to evaluate the response of rats on noradrenaline (NA)-induced contractions.

**Results:**

The rats administered LBPs showed longer swimming time until exhaustion than the control group of rats. Exercise-induced MDA elevation was repressed by LBPs supplementation. The LBPs-supplemented rats displayed a significant increase of SOD, NO, HSP70 than the non-supplemented rats. Additionally, LBPs significantly up-regulated the expression of eNOS and improved the endothelium-dependent vasodilatation of the aorta ring.

**Conclusion:**

Our study proved that LBPs administration significantly inhibited the oxidative stress, and improved the arterial compliance.

## Introduction

Arterial compliance, the inverse of arterial stiffness, is now recognized as an important determinant of cardiovascular morbidity and mortality [1]. Exercise can affect arterial compliance. It is well known that aerobic exercise reduces arterial stiffness. Moderate-intensity aerobic exercise at 65% of its maximal oxygen uptake lowers both central and peripheral arterial stiffness [2]. In addition, twelve weeks of aerobic exercise enhances vascular compliance (especially of the arms and legs) in obese male adolescents [3]. However, the beneficial effects of exercise are lost with exhaustion. For example, High-intensity strength exercise leads to a decrease in arterial compliance [4,5]. Twenty to forty hours of continuous mountain trail running decreases the large artery compliance [6]. Moreover, marathon runners have increased aortic stiffness compared to that of the control group [7]. In contrast, one-year of exercise fails to improve the arterial stiffness or function of heart failure with preserved ejection fraction (HFpEF) in patients [8]. The mechanism of different effects of exercise on arterial compliance remains unclear.

Lycium barbarum (also called Wolfberry, Fructus Lycii or Gouqizi), belonging to the plant family Solanaceae, has been widely used for 2000 years in traditional Chinese Medicine [9-11]. Polysaccharides (LBPs) which constitute more than 40% of the fruit extract are the major valuable and active ingredient in Lycium barbarum [12]. LBPs have been shown to exert a large variety of biological activities including eye-protective, anti-aging, antioxidant, immunoregulating, neuroprotective, cytoprotective and antitumor properties [13-17]. It has been reported that LBPs treatment prevented the increase of blood pressure in hypertension rats induced by the two-kidney, one clip method in vivo. LBPs-treated rats showed a significant decrease in the concentration of phenylephrine in isolated aortic rings as compared with non-treated hypertensive rats [18]. However, the effects of LBPs on arterial compliance in rats with exhaustive exercise have not been investigated. In the present study, we aimed to determine the effects of LBPs on the arterial compliance from lesions induced by exhaustive exercise.

## Materials and methods

### Animals

A total of 40 male Sprague Dawley rats (180 ± 20 g) were bred, five per cage, in light-and temperature-controlled conditions (12 hours light: 12 hours dark; 24.0 ± 0.2°C) and provided with standard laboratory diet and tap water ad libitum. The experimental procedures were approved by the animal ethics committee of the Ningxia Medical University and Use Committee in accordance with the guidelines of the Council of the Physiological Society of China.

After an adaptation period of one week, all animals were randomly divided into 4 groups (n = 10): control sedentary group (CS), swimming exercise group (SE), exhaustive swimming exercise group (ES), exhaustive swimming exercise with LBPs group (ES-LBP). The rats in ES-LBP group received 200 mg/kg/day by gavage for 28 days. In CS, SE, ES groups, the rats were given the same volume of isotonic saline solution by oral administration for 28 days. The dose of LBPs was chosen on the basis of preliminary experiments, which was safe and effective without undue toxicity in rats.

### Exercise protocol

During the first week, rats were acclimated to swimming exercises for 5 days with increasing duration from 5 minutes on the first day to 60 minutes by the fifth day [19]. The rats in the control group were subjected to water immersion without exercises. The rats swam in a plastic tank (diameter, 60 cm; depth, 80 cm) filled with water at 32 ±1°C. After acclimation, rats were assigned to swim for 60 minutes per day, 5 days per week, for 4 weeks (between 8:00 am and 12:00 am). At the end of the training, the rats of the ES and ES-LBP groups were subjected to a swim to exhaustion with a load of 5% of their body weight strapped on their backs. The point of exhaustion was defined when a rat failed to rise to the surface of water, drown over 10 seconds and could not maintain coordination [20]. This exhaustion time was subsequently recorded.

### Samples collection

All animals were anesthetized with urethane (1.5 g/kg) and sacrificed immediately after the exhaustive exercise. The chest was rapidly opened and the thoracic aorta was carefully isolated in order to preserve the vascular endothelium, which was then placed into modified cold Krebs’ solution. The isolated vessel was cut into rings of approximately 3–4 mm wide for measuring isometric force. The rest of the aorta was frozen in liquid nitrogen immediately and stored at -80°C for the assay of endothelial NO synthase (eNOS) mRNA expression . Blood was collected from inferior vena cava in heparinized tube and centrifuged at 1,700×g for 10 minutes (at 4°C) to obtain plasma. The plasma was frozen at -80°C to measure the expression of malondialdehyde (MDA), super oxide dismutase (SOD), nitric oxide (NO) and heat shock protein 70(HSP70).

### Assay of isometric force in Rat aorta rings

The isolated aortic rings were cleaned to remove the adherent tissues and hung in 10-ml organ bath with Krebs’ solution at 37°C, pH 7.4, and containing 95% O_2_ and 5% CO_2_. The modified Krebs’ solution was composed of the following components: 110 mM NaCl, 4.6 mM KCl, 2.5 mM CaCl_2_, 24.8 mM NaHCO_3_, 1.2 mM KH_2_PO_4_, 1.2 mM MgSO_4_, and 5.6 g glucose. The tissue’s isometric tension was measured with force transducers that connected with a BL-420E^+^ biological function experimental system (Chengdu Technology and Market, Chengdu, China). The vessel rings were equilibrated for 1 hour with the tension of 2.0 g and pre-contracted with KCl (60 mM) to produce the maximal KCL-induced contractile plateau. Subsequently the cumulative dose–response curve for noradrenaline (NA) (10^-10^-10^-5^M) was obtained. The values of the NA-induced contraction were expressed as a percentage of maximal contraction induced by KCl.

### Measurement of SOD, MDA and nitrite/nitrate (NOx) levels in plasma

The oxidative stress indices were measured to explore whether LBP could reduce exhaustive exercise-induced oxidative stress. The levels of SOD, MDA and NOx (NO^2-^ and NO^3-^) were determined by using commercially available kits (Nanjing Jiancheng Bioengineering Institute, Nanjing, China) according to the manufacturer’s instructions.

### HSP70 determination

The plasma level of HSP70 was detected by a commercially available ELISA kit (Cusabio Biotechnology, Wuhan, China). The amount of HSP70 in plasma was estimated from the calibration curve ranging from 62.5 to 4000 pg/ml.

### RT-PCR analysis

Total RNA was prepared from the thoracic aorta using RNA AxyPrep Pure RNA isolation kit (AXYGEN, USA) according to the manufacturer’s instructions. The purity and concentration of RNA was determined by spectrophotometry at 260 nm and 280 nm. Complementary DNA (cDNA) was synthesized using a reverse transcription kit (TransGen Biotechnology, Beijing). Quantitative PCR was performed using a quantitect SYBR green PCR kit (TransGen Biotechnology, Beijing) as follows: 35 cycles of denaturation at 94°C for 30 sec, annealing at 62°C for 30 sec and extension at 72°C for 30 sec. Primers used for the PCR were shown in Table 1. Relative gene expression levels were determined using the 2^—△△Ct^ method.

**Table 1:** GenBank accession code, primer sequences, and predicted size of the amplified product

**Gene**	**Primer sequences**	**GenBank**	**bp**
eNOS	Forward primer: 5′-CACACTGCTAGAGGTGCTGGAA-3′	NM_021838	109
	Reverse primer: 5′-TGCTGAGCTGACAGAGTAGTAC-3′		
β-actin	Forward primer: 5′-TCATGAAGTGTGACGTTGACATCCGT-3′		285
	Reverse primer: 5′-CCTAGAAGCATTTGCGGTGCAGGATG-3′		

### Statistical analysis

Results were presented as the mean ± SD. Two-way ANOVA was used to evaluate any differences between the two sets of dose–response curves. The remaining data were evaluated by one-way ANOVA and Student’s t-test. The statistical analyses were performed by SPSS for Windows 11.5.0 software. P<0.05 was considered to indicate a statistically significant difference.

## Results

### Contractile response of vascular ring to NA

**Figure 1:**
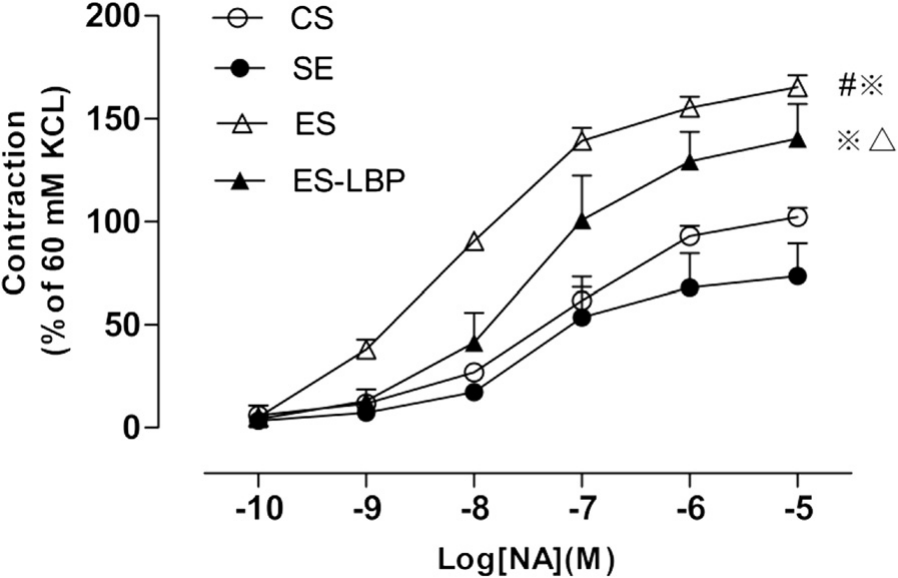
**Contractile response of vascular ring to NA.** Dose-dependence of NA on contraction of the thoracic aorta rings separated from rats in CS SE, ES and ES-LBP groups. The contraction induced by 60 mM KCl was taken as 100%. Data are expressed as mean ± SD (*n*=10). ^#^*P*<0.01 *vs* CS; ^※^*P*<0.01*vs* SE; ^△^*P*<0.01 *vs* ES.

Vascular dysfunction is related to increased vasoconstriction and weakened diastolic function. Therefore, we are interested in determining whether there is any change in the vascular function by detecting the vascular reactivity of aortic rings to a physiological modulator, noradrenaline (NA). Cumulatively added NA (10^-10^-10^-5^M) caused concentration-dependent contractile responses in isolated aortic rings. We found that there was no significant difference between the SE and the CS group, while the ES group significantly increased the vasoconstrictive response to NA (*P*<0.01), LBPs treatment decreased the vasoconstrictive effect ( *P*< 0.01) (Figure 1). Furthermore, the contractile responsiveness to NA of the SE group was significantly lower than that of the ES (*P*<0.01) and ES-LBP (*P*<0.01) groups (Figure 1).

### Effects of LBPs on body weight and exhaustive exercise time in rats

**Figure 2:**
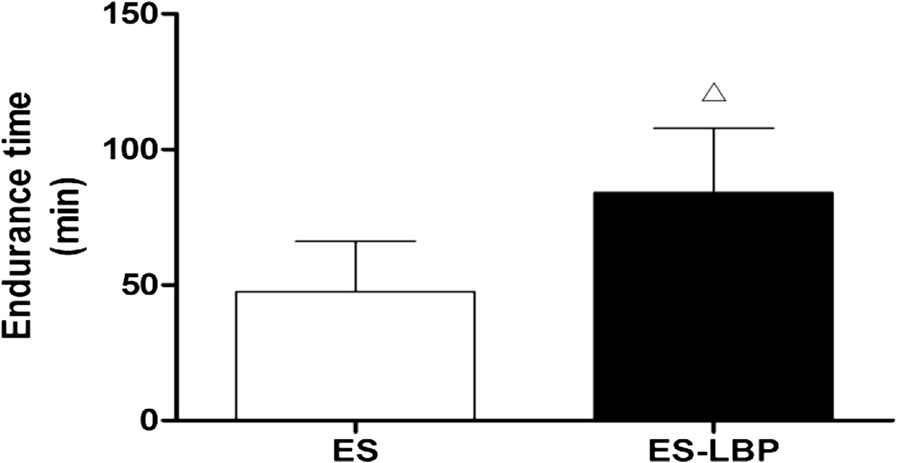
**Effects of LBPs on exhaustive exercise time in the rats.** LBPs supplementation significantly increased the time to fatigue compared to that of the ES. Data are mean ± SD (*n* =10). ^△^*P* < 0.01 *vs* ES.

After four weeks of swimming exercise, no significant difference was observed in body weight in either group (Table 2). However, as shown in Figure 2, LBPs prolonged the swimming time of rats compared with the ES group ( *P* < 0.05), which was 77.07% higher.

**Table 2:** Effects of LBP on body weight in rats

**Group**	**Before experiment**	**One week**	**Two week**	**Three week**	**Four week**
CS	191.67±26.90	204.83±13.43	264.08±12.31	304.44±9.97	346.58±15.55
SE	187.5±4.74	209.53±6.15	258.43±9.88	309.35±19.11	340.5±22.31
ES	191.2±10.77	210.67±10.91	263.5±14.05	304.58±17.12	329.13±15.06
ES-LBP	198.2±9.66	215.14±7.22	267.70±6.96	312.08±10.14	344.33±14.91

Effects of LBPs on body weight in rats. The values are expressed as mean ± SD (*n*=10).

### Effects of LBPs on biochemical parameters after exhaustive exercise

**Figure 3:**
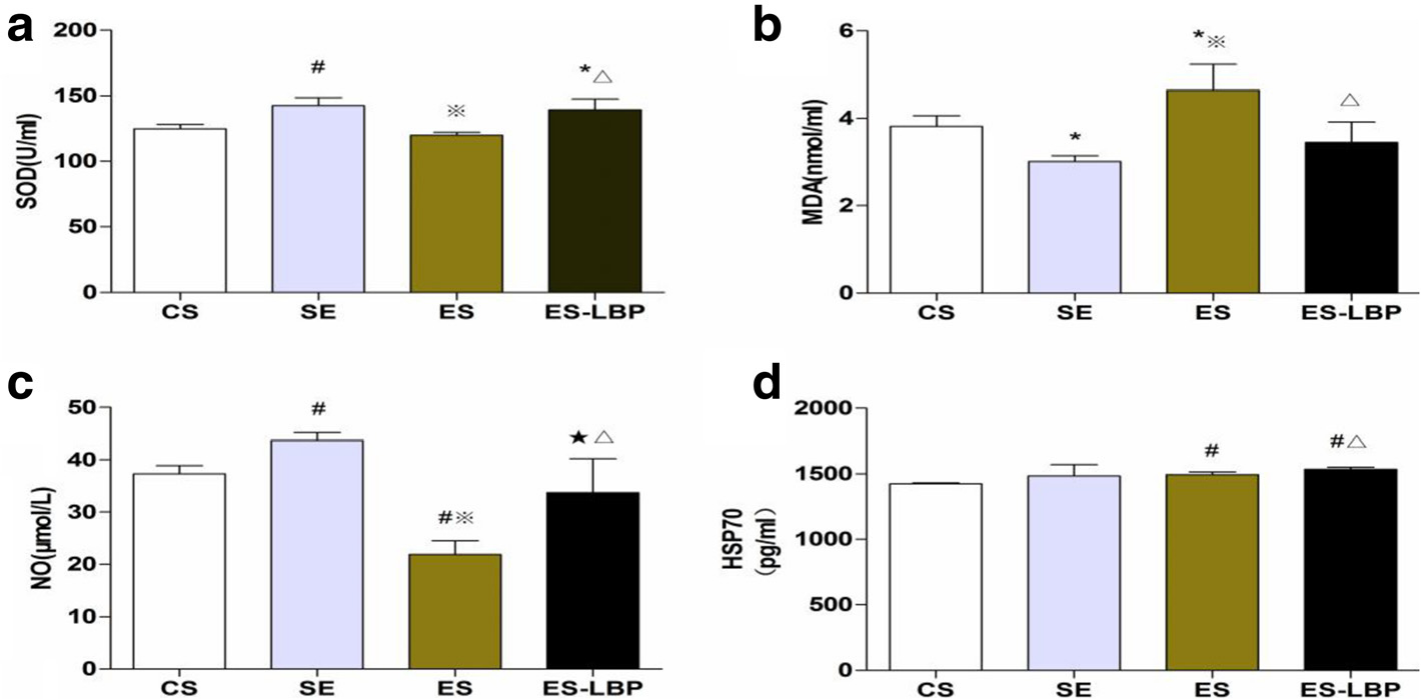
**Effects of LBPs supplement and exhaustive exercise on SOD (a), MDA (b), NO (c) and HSP70 (d) expression in the rats.** Values are expressed as mean ± SD (*n* = 10). *P < 0.05 and ^#^*P* < 0.01 *vs* CS; ^★^*P* < 0.05 and ^※^< 0.01 *vs* SE; ^△^*P* < 0.01 *vs* ES.

It is well known that SOD can inhibit the oxidation of oxyamine by the xanthine–xanthine oxidase system. Therefore we evaluated the plasmic level of SOD. As shown in Figure 3a, the SOD level in the ES-LBP, SE groups significantly increased compared with that in the CS group (*P*<0.05 and *P*<0.01 respectively). However, the plasmic SOD level of exhaustive swimming rats was significantly lower than that of the ES-LBP and SE rats (*P*< 0.01). The results demonstrated that LBPs were able to increase antioxidant enzyme activities to attenuate the oxidative stress induced by exhaustive exercise.

Exhaustive exercise induces the generation of free radicals which may cause an increase in lipid peroxidation [21]. Measuring MDA is one of the most widely used approaches for evaluating oxidative damage to lipids. Figure 3b illustrates that the plasmic MDA levels of SE or ES-LBP rats significantly decreased compared with that of ES rats (*P*<0.05 and *P*< 0.01 respectively). This result indicates that LBPs can attenuate lipid peroxidation.

NO is an important vasodiator factor produced by vascular endothelial cells. We found that there was a significant increase in the SE group. As expected, the NO level was significantly reduced by exhaustive exercise. Further, we found this reduction induced by exhaustive exercise could be reversed by LBPs treatment (Figure 3c).

The expression of heat shock proteins (HSPs) is induced by hyperthermia ischemia, oxidative cytokine, muscular stress, glucose deprivation, alterations in calcium and pH [22]. HSP70 is a group of binding proteins with molecular weight of 70 KD, which is significantly increased by high-intensity exercise [23]. To determine the expression of HSP70 after exercise and supplement with LBPs, the plasmic level of HSP70, analyzed by ELISA, showed an immediate increase after both exercise sessions. As shown in Figure 3d, the HSP70 levels of SE or ES rats were increased. Furthermore, LBPs treatment induced a much higher increase in the ES group (*P*< 0.01).

### Expression of eNOS mRNA

**Figure 4:**
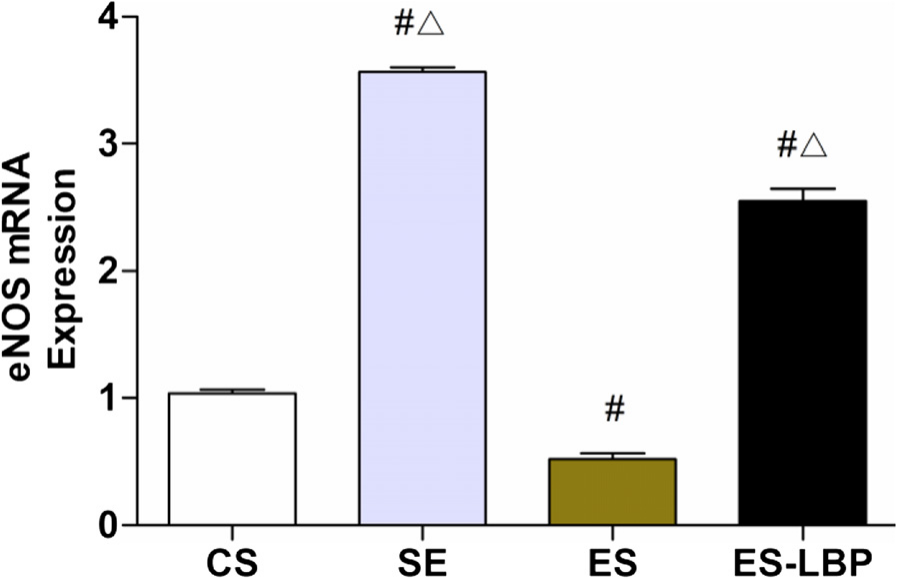
**Effects of LBPs on eNOS mRNA expression in thoracic aorta separated from rats in different groups.** Values are expressed as mean ± SD (*n* = 10). ^#^*P*<0.01 *vs* CS; ^△^*P*<0.01 *vs* ES.

As the NO level can be up-regulated by LBPs, we therefore examined the effect of LBPs on the expression of eNOS in the aorta after exhaustive exercise. The expression of eNOS mRNA in aorta of four groups was shown in Figure 4. There were significant differences in the eNOS mRNA expression level among different groups. The eNOS expression was increased in both SE and ES-LBP groups (*P* < 0.01). However, the level of eNOS expression was significantly attenuated in rats after exhaustive exercise (*P* < 0.01). LBPs treatment significantly reversed the inhibition of the eNOS expression in rats from ES group (*p* < 0.01).

## Discussion

The effects of LBPs on vascular vasoreactivity in exhaustive exercise rats were investigated. The major finding of this study was that the contraction induced by NA in thoracic aorta was increased in the presence of exhaustive exercise. Furthermore, supplementation with the LBPs for 4 weeks remarkably improved the vascular reactivity of ES-LBP rats compared to the ES rats (Figure 1). As the arterial compliance is judged by the responsiveness to NA, the results showed that the compliance or distensibility of aorta was increased in LBPs treated animals [24].

Arterial compliance, the inverse of arterial stiffness, is now recognized as an important determinant of cardiovascular morbidity and mortality [1], which can increase the workload placed on the myocardium. The changes in arterial compliance of exercise training rats depend on the exercise mode, intensity and duration. Twelve weeks of air board exercise leads to an increase in cardio-respiratory fitness and vascular compliance, which may reduce the risk of later development of cardiovascular disease [3] and improve coronary artery perfusion preventing ischemic events [25], and decline pulse pressure and wall stress [26]. Moreover, Nickel [27] showed that 30 minutes of moderate-intensity aerobic exercise transiently increased small arterial compliance after exercise, but not sustained. Extremely high volume exercise may be associated with decreases in cardiovascular function and large artery compliance [6]. Ahmadi et al. [28] recently reported that coronary artery calcification was associated with impaired aortic compliance.

The present study has confirmed these varying effects of exercise on arterial compliance. In SE rats, which were subjected to swimming exercise for four weeks, the attenuated contractile responses of aorta to NA were clearly observed, whereas in rats exposed to exhaustive swimming exercise, depressed vasodilator response was observed (Figure 1). This inhibition was completely reversed by the treatment of LBPs in the ES group. In isolated aortic rings of LBPs-treated rats, the responsiveness to phenylephrine was attenuated in comparison with non-treated hypertensive rats [18].

Generally, exhaustive exercise induced oxidative stress impaired endothelial function [29] that decreased artery compliance [30], which may interfere with NA-dependent vasoconstriction. The present study indicated that a bout of exhaustive swimming exercise caused a significant increase in oxidative stress, which decreased the serum antioxidant enzyme SOD and increased the lipid hydroperoxides MDA. LBPs were shown to be effective in avoiding oxidative stress and cleaning out the excess free radical and decreasing the level of lipid peroxidation [10,31,32]. These increases in super oxide levels were correlated with attenuated responsiveness to NA. Our previous study also showed that LBPs could enhance the immune function in exhausted swimming rat [33]. Combination with results of this study, LBPs is a useful protective agent in rats of exhaustive exercise, and whether LBPs are helpful for athletes needs a further research to confirm.

NO, derived from a biochemical reaction catalyzed by eNOS [34], plays an important role in the regulation of vascular tension [35]. The most important activity of NO may be vasodilation in the cardiovascular system, which is usually used as a surrogate index of endothelial function [35]. Studies have demonstrated that arterial stiffness was regulated by the endothelium through the release of NO [36]. Our data showed that LBPs could enhance the expression of eNOS, elevate NO levels and consequently inhibit the contractile response to NA. Previous studies have suggested that N-nitro-L-arginine methyl ester increased the contraction to phenylephrine in the aortic rings of LBPs-treated rats in vitro. LBPs reduced the phenylephrine-induced contraction which may be mediated by increasing the production of endothelium-derived relaxation factor (EDRF) [18]. In addition, aortic contractility of LBPs-treated rats reduced due to attenuated responsiveness to NA and probably to increase in plasmic level of NO. The up-regulation of SOD levels during exercise training might lead to improvement in endothelial function through an increase in NO production [37].

Heat shock proteins (HSP) belong to the family of stress-responsive proteins that are induced by oxidative stress, which are essential for modulating cell function and maintaining protein homeostasis [38,39]. As a stress protein, the response of HSP70 is different according to the intensity and form of movement, which provides new ideas and methods to further understand the campaign laws and institute more scientific physical training and exercise training [40,41]. In ES-LBP, the HSP70 levels were significantly increased compared with that of ES. Meanwhile, the attenuation of the NA-induced aortic contraction was observed in ES-LBP rats. Thus, HSP70 may take part in this attenuation through protecting the cells from the deleterious effects of ROS and reducing oxidative stress.

## Conclusion

In conclusion, this study clearly indicates that the contractile response to NA is attenuated by LBPs treatment in ES-LBP rats. The exhaustive swim time is also prolonged by LBPs supplement through activation of the antioxidant defense system. Meanwhile, LBPs can up-regulate the expression of eNOS, NO and HSP70. However, the mechanism of blunted contractile response to NA in aorta of LBPs-treated rats is not fully investigated in this study, further research including molecular study is required to investigate this mechanism.

## Competing interests

The authors declare that they have no competing interests.

## Authors’ contributions

GL: dissertation guidance, interpretation of the data and and drafted the manuscript; ZZ: randomization of the protocol training of animals, literature review; YL: molecular biology assays; LZ: ELISA assays assistance and biochemical assays; YW: paper revise; XZ: animal training assistance; All authors read and approved the final manuscript.

## References

[B1] Cavalcante JL Lima JAC Redheuil A Aortic stiffness current understanding and future directions J Am Coll Cardiol 2011 10 14 1511 1522 10.1016/j.jacc.2010.12.017 21453829

[B2] Heffernan K Collier S Kelly E Arterial stiffness and baroreflex sensitivity following bouts of aerobic and resistance exercise Int J Sports Med 2007 10 3 197 10.1055/s-2006-924290 17024636

[B3] Song JK Stebbins CL Kim TK Effects of 12 weeks of aerobic exercise on body composition and vascular compliance in obese boys J Sports Med Phys Fitness 2012 10 5 522 529 22976739

[B4] Otsuki T Maeda S Iemitsu M Vascular endothelium-derived factors and arterial stiffness in strength-and endurance-trained men American J Physiol-Heart Circ Physiol 2007 10 2 H786 H791 10.1152/ajpheart.00678.2006 16997889

[B5] Kawano H Tanimoto M Yamamoto K Resistance training in men is associated with increased arterial stiffness and blood pressure but does not adversely affect endothelial function as measured by arterial reactivity to the cold pressor test Exp Physiol 2008 10 2 296 302 17911355 10.1113/expphysiol.2007.039867

[B6] Burr J Bredin SS Phillips A Systemic arterial compliance following ultra-marathon Int J Sports Med 2012 10 03 224 229 22261822 10.1055/s-0031-1297956

[B7] Vlachopoulos C Kardara D Anastasakis A Arterial stiffness and wave reflections in marathon runners Am J Hypertens 2010 10 9 974 979 10.1038/ajh.2010.99 20489686

[B8] Fujimoto N Prasad A Hastings JL Cardiovascular effects of 1 year of progressive endurance exercise training in patients with heart failure with preserved ejection fraction Am Heart J 2012 10 6 869 877 10.1016/j.ahj.2012.06.028 23194487 PMC3727249

[B9] Deli MA Yang D Li S-Y Lycium barbarum extracts protect the brain from blood–brain barrier disruption and cerebral edema in experimental stroke Plos One 2012 10 3 e33596 10.1371/journal.pone.0033596 22438957 PMC3306421

[B10] Shan XZ Zhou JL Ma T Lycium barbarum polysaccharides reduce exercise-induced oxidative stress Int J Mol Sci 2011 10 2 1081 1088 21541044 10.3390/ijms12021081 PMC3083691

[B11] Potterat OG (Lycium barbarumandL. chinense): phytochemistry, pharmacology and safety in the perspective of traditional uses and recent popularity Planta Med 2009 10 01 7 19 19844860 10.1055/s-0029-1186218

[B12] Chang RC-C So K-F Use of anti-aging herbal medicine, lycium barbarum, against aging-associated diseases. What do we know so far? Cell Mol Neurobiol 2007 10 5 643 652 17710531 10.1007/s10571-007-9181-x PMC11514989

[B13] Ho YS Yu MS Yik SY Polysaccharides from wolfberry antagonizes glutamate excitotoxicity in rat cortical neurons Cell Mol Neurobiol 2009 10 8 1233 1244 10.1007/s10571-009-9419-x 19499323 PMC11505788

[B14] Wu HT He XJ Hong YK Chemical characterization of Lycium barbarum polysaccharides and its inhibition against liver oxidative injury of high-fat mice Int J Biol Macromol 2010 10 5 540 10.1016/j.ijbiomac.2010.02.010 20193709

[B15] Tang W-M Chan E Kwok C-Y A review of the anticancer and immunomodulatory effects of Lycium barbarum fruit Inflammopharmacology 2012 10 6 14 307 10.1007/s10787-011-0107-3 22189914

[B16] Chang HM But PPH Yao SC Pharmacology and applications of Chinese materia medica 2001 Singapore: World Scientific Publishing Company Incorporated

[B17] Potterat O Phytochemistry, pharmacology and safety in the perspective of traditional uses and recent popularity Planta Med 2010 10 7 19 10.1055/s-0029-1186218 19844860

[B18] Jia YX Dong JW Wu XX The effect of lycium barbarum polysaccharide on vascular tension in two-kidney, one clip model of hypertension Sheng Li Xue Bao 1998 10 3 309 314 11324572

[B19] Sampaio-Barros M Farias-Silva E Grassi-Kassisse D Effect of swimming session duration and repetition on metabolic markers in rats Stress: Int J Biol Stress 2003 10 2 127 132 10.1080/1025389031000110169 12775332

[B20] Thomas D Marshall K Effects of repeated exhaustive exercise on myocardial subcellular membrane structures Int J Sports Med 1988 10 4 257 260 10.1055/s-2007-1025017 3182155

[B21] Skarpańska-Stejnborn A Basta P Pilaczyńska-Szcześniak Ł Black grape extract supplementation attenuates blood oxidative stress in response to acute exercise Biol Sport 2010 10 1 41 46

[B22] Fehrenbach E Northoff H Free radicals, exercise, apoptosis, and heat shock proteins Exerc Immunol Rev 2001 10 66 89 11579749

[B23] Noble EG Moraska A Mazzeo RS Roth DA Olsson MC Moore RL Fleshner M Differential expression of stress proteins in rat myocardium after free wheel or treadmill run training J Appl Physiol 1999 10 5 1696 701 10233137 10.1152/jappl.1999.86.5.1696

[B24] Dunbar SL Tamhidi L Berkowitz DE Hindlimb unweighting affects rat vascular capacitance function American J Physiol-Heart Circ Physiol 2001 10 3 H1170 H1177 10.1152/ajpheart.2001.281.3.H1170 11514284

[B25] Kass DA Saeki A Tunin RS Adverse influence of systemic vascular stiffening on cardiac dysfunction and adaptation to acute coronary occlusion Circulation 1996 10 8 1533 1541 10.1161/01.CIR.93.8.1533 8608622

[B26] Belz GG Elastic properties and Windkessel function of the human aorta Cardiovasc Drugs Ther 1995 10 1 73 83 10.1007/BF00877747 7786838

[B27] Nickel KJ Acree LS Gardner AW Effects of a single bout of exercise on arterial compliance in older adults Angiology 2011 10 1 33 37 10.1177/0003319710381993 21134994 PMC3076948

[B28] Ahmadi N Nabavi V Hajsadeghi F Impaired aortic distensibility measured by computed tomography is associated with the severity of coronary artery disease Int J Cardiovasc Imaging (formerly Cardiac Imaging) 2011 10 3 459 469 10.1007/s10554-010-9680-6 PMC3092065 20711815

[B29] Knez WL Coombes JS Jenkins DG Ultra-endurance exercise and oxidative damage: implications for cardiovascular health Sports Med 2006 10 5 429 441 10.2165/00007256-200636050-00005 16646630

[B30] Wilkinson IB MacCallum H Cockcroft JR Inhibition of basal nitric oxide synthesis increases aortic augmentation index and pulse wave velocity in vivo Br J Clin Pharmacol 2002 10 2 189 192 10.1046/j.1365-2125.2002.1528adoc.x 11851643 PMC1874288

[B31] Niu A-J Wu J-M Yu D-H Protective effect of Lycium barbarum polysaccharides on oxidative damage in skeletal muscle of exhaustive exercise rats Int J Biol Macromol 2008 10 5 447 449 10.1016/j.ijbiomac.2008.02.003 18405964

[B32] Duan CB Sun ZJ Supplementation of Lycium barbarum polysaccharides protection of skeletal muscle from exercise-induced oxidant stress in mice African J Pharm Pharmacol 2012 10 9 643 647

[B33] Jing H Hong-peng L Zhou X Guang-hua L The effect of LBP on immune function of exhausted swimming exercise in mice J Liaoning University of TCM 2009 10 8 234 236

[B34] Li GH Katakura M Maruyama M Changes of noradrenaline-induced contractility and gene expression in aorta of rats acclimated to heat in two different modes Eur J Appl Physiol 2008 10 1 29 40 10.1007/s00421-008-0772-0 18512069

[B35] Maiorana A O’Driscoll G Taylor R Exercise and the nitric oxide vasodilator system Sports Med 2003 10 14 1013 1035 10.2165/00007256-200333140-00001 14599231

[B36] Bellien J Favre J Iacob M Arterial stiffness is regulated by nitric oxide and endothelium-derived hyperpolarizing factor during changes in blood flow in humans Hypertension 2010 10 3 674 680 10.1161/HYPERTENSIONAHA.109.142190 20083732

[B37] Higashi Y Yoshizumi M Exercise and endothelial function: role of endothelium-derived nitric oxide and oxidative stress in healthy subjects and hypertensive patients Pharmacol Ther 2004 10 1 87 96 10.1016/j.pharmthera.2004.02.003 15056500

[B38] Asea A Hsp70: a chaperokine Novartis Foundation symposium; 2008 2008 10 Chichester; New York: John Wiley 173 10.1002/9780470754030.ch13 18575273

[B39] Atalay M Oksala N Lappalainen J Heat shock proteins in diabetes and wound healing Curr Protein Pept Sci 2009 10 1 85 10.2174/138920309787315202 19275675 PMC2743605

[B40] Banfi G Dolci A Verna R Exercise raises serum heat-shock protein 70 (Hsp70) levels Clin Chem Lab Med 2004 10 12 1445 1446 15576310 10.1515/CCLM.2004.268

[B41] Guixia C Junwei B Progress of the research on the effect of exercises on HSP70 expression in cardiac and skeletal muscles J Jilin Institute of Phys Educ 2010 10 5 83 85

